# Effect on Hemoglobin A1c (HbA1c) and Body Weight After Discontinuation of Tirzepatide, a Novel Glucose-Dependent Insulinotropic Peptide (GIP) and Glucagon-Like Peptide-1 (GLP-1) Receptor Agonist: A Single-Center Case Series Study

**DOI:** 10.7759/cureus.46490

**Published:** 2023-10-04

**Authors:** Mitsunobu Kubota, Kazuki Yamamoto, Sayo Yoshiyama

**Affiliations:** 1 Department of Endocrinology and Diabetes, National Hospital Organization, Kure Medical Center and Chugoku Cancer Center, Kure, JPN

**Keywords:** body weight, type 2 diabetes, hba1c, gip and glp-1 receptor agonist, tirzepatide

## Abstract

Introduction

The purpose of this study was to examine changes in blood glucose levels and body weight after discontinuation of tirzepatide, a novel long-acting dual glucose-dependent insulinotropic peptide (GIP) and glucagon-like peptide-1 receptor agonist (GLP-1 RA).

Methods

Nine subjects (five males, four females, age 54.3±5.4 years, body mass index 33.5±3.3 kg/m^2^) participating with type 2 diabetes in the SURPASS J-mono study were included. Subjects were randomized to tirzepatide 5 mg, 10 mg, 15 mg, or a dulaglutide 0.75 mg group. Fifty-two weeks after randomization, study drug administration was discontinued. To investigate progress after the end of administration, changes in hemoglobin A1c (HbA1c) and body weight were further examined two, four, and six months after discontinuation of the study drug.

Results

After fifty-two weeks, all tirzepatide groups had improved HbA1c and body weight compared with the dulaglutide group. At two, four, and six months after the end of study drug administration, re-elevation of HbA1c was observed in all groups. Furthermore, in the tirzepatide groups, dose-dependent weight regain was observed from an early stage.

Conclusions

Compared to dulaglutide, tirzepatide exhibited excellent blood-glucose-improving and weight-reducing effects. However, exacerbation of blood glucose and rebound of weight gain occurred relatively early after administration was ended. For type 2 diabetes patients who need weight loss and are prescribed tirzepatide, these findings suggest a necessity for continuous prescription or careful follow-up when stopping.

## Introduction

Type 2 diabetes is on the rise worldwide [1]. In patients with type 2 diabetes, improved glycemic control contributes to reduced risk of complications such as microangiopathy, myocardial infarction, and stroke [2], and lower hemoglobin A1c (HbA1c) may lead to improved life prognosis [3]. In Japanese patients, it has been reported that intensive control of not only blood glucose but also blood pressure and lipid metabolism contributes to the improvement of diabetic complications [4]. In recent years, it has recently been reported that the body mass index (BMI) of type 2 diabetes patients tends to increase, and the complication rate of obesity is increasing, especially among young people [5]. The DiRECT study evaluated the relationship between weight loss and type 2 diabetes remission rates, and approximately one-quarter of people with type 2 diabetes were close to remission after losing weight in the first year of the study [6]. It has been reported that insulin resistance is significantly higher in Japanese with obesity [7], and fatty liver associated with obesity causes insulin resistance, which leads to glucose intolerance. Through these mechanisms, the remission rate of diabetes decreases in fatty liver associated with obesity [8]. Moreover, fat accumulation in the liver and pancreas, which is associated with excess caloric intake and increased insulin resistance, has been suggested to cause type 2 diabetes [9]. In order to maintain good glycemic control, it is important to reduce visceral fat mass and improve insulin resistance. As the number of patients with type 2 diabetes mellitus combined with obesity increases, controlling not only blood glucose but also body weight becomes an urgent issue.

Glucose-dependent insulinotropic peptide (GIP) and glucagon-like peptide-1 (GLP-1), the incretin hormones secreted from the small intestine, play major roles in postprandial insulin secretion [10], with GIP reportedly having the greater effect in subjects without type 2 diabetes [11]. GLP-1 has already been clinically applied as an antidiabetic agent and is expected to reduce postprandial blood glucose fluctuations and weight loss by stimulating insulin secretion, suppressing excessive secretion of glucagon, and delaying gastric excretion [12]. Similar to GLP-1, GIP enhances glucose-dependent insulin secretion, but its action is attenuated by GIP resistance in chronic hyperglycemia [13,14] and may be restored by correction of hyperglycemia [15]. Regarding effects on body weight, it was reported that at physiological concentrations GIP promotes fat accumulation in a mouse model [16], but at pharmacological concentrations, it suppresses appetite and reduces body weight [17].

Tirzepatide, a 39-amino-acid synthetic peptide designed from the GIP sequence, is a novel long-acting dual GIP/GLP-1 receptor agonist. It contains a C20 fatty acid diacid moiety, has an extended half-life (about 5 days), and can be administered subcutaneously once a week [18]. The global SURPASS-2 trial confirmed that once-weekly doses of tirzepatide (5, 10, and 15 mg) have a clinically meaningful blood glucose improvement effect relative to the conventional GLP-1 receptor agonist semaglutide (1 mg) [19]. In addition, the SURPASS J-mono trial [20] and the SURPASS J-combo trial [21], which were also conducted in Japanese patients, demonstrated tirzepatide to improve glycemic control and reduce body weight compared with once-weekly administration of 0.75 mg dulaglutide, a GLP-1 receptor agonist that is frequently used in clinical settings. In particular, 90% or more of patients achieved HbA1c of less than 6.5% in all groups receiving tirzepatide, which is extremely high compared to conventional antidiabetic drugs.

The SURPASS-1 trial disclosed that fasting blood glucose levels rebounded to 20.2 mg/dL in the tirzepatide 5 mg group, 22.5 mg/dL in the 10 mg group, and 17.8 mg/dL in the 15 mg group over a four-week follow-up period after the end of tirzepatide administration [22]. After discontinuing treatment with liraglutide, a GLP-1 receptor agonist, blood glucose control and body weight worsened compared to those who continued treatment [23]. The mechanisms by which blood glucose and body weight rebound upon discontinuation of GLP-1 receptor agonists are presumed to be the cancellation of its appetite-suppressing effect, the disappearance of gastrointestinal symptoms such as nausea and vomiting, and the cancellation of the stimulation of glucose-dependent insulin secretion [23]. However, it is not yet known to what extent discontinuation of tirzepatide affects blood glucose and body weight chronologically in the overall SURPASS J-mono trial. The clinical trial follows a protocol to uniformly discontinue the study drug after the end of the study period, so it is appropriate to investigate post-continuation changes among trial participants. Therefore, in this study, we aimed to investigate the effects of tirzepatide on blood glucose and body weight after terminating administration in patients with type 2 diabetes who participated in the SURPASS J-mono trial [20].

## Materials and methods

Study procedure

The SURPASS J-mono trial is a multicenter, randomized, double-blind, parallel, active-controlled phase 3 trial of tirzepatide conducted at 46 medical centers and hospitals in Japan [20]. Please refer to the SURPASS J-mono study for the details of study inclusion and exclusion criteria [20]. The SURPASS J-mono study was approved in our hospital by the Ethics Committee of National Hospital Organization of Kure Medical Center (Approval no. 2019-01). This study was conducted independently of the overall analysis of the SURPASS J-mono study as a case series study of nine patients and an observational study examining the effect of withdrawing the study drug. This study was conducted in accordance with the Declaration of Helsinki and International Ethical Guidelines by the Council for International Organizations of Medical Sciences. This particular study that included nine patients and investigated the progress after tirzepatide administration after two, four, and six months was approved by the Ethics Committee of National Hospital Organization of Kure Medical Center (Approval no. 29-93) and all participants provided written informed consent.

Study design

Participants were randomly assigned by the investigator to receive tirzepatide (5, 10, or 15 mg) or dulaglutide (0.75 mg) using a computer-generated random sequence with an Interactive Web Response System. Subcutaneous injections of tirzepatide or dulaglutide were administered weekly for 52 weeks. The starting dose of tirzepatide was 2.5 mg, and the dose was increased by 2.5 mg every four weeks until reaching the target dose. The dulaglutide group received 0.75 mg weekly. All study drugs were administered in a standardized manner using disposable pens with the same injection volume (0.5 mL).

Study group enrollment

Of the 13 patients with type 2 diabetes at our department who were screened for eligibility for the SURPASS J-mono trial, 1 became ineligible due to improved control during the period before administration of the study drug, 2 became ineligible due to worsening glycemic control, and 1 withdrew consent. The remaining 9 subjects received the study drug and were included in the analysis. These subjects comprised 5 men and 4 women with an average age of 54.3 ± 5.4 years (mean ± standard error). Consent for study inclusion was obtained between June 19, 2019 and February 21, 2020.

Study outcome

The primary endpoint of the SURPASS J-mono study was the mean change in HbA1c from baseline at week 52 measured in the modified intention-to-treat population. Furthermore, in order to evaluate the therapeutic effect after the end of tirzepatide administration, changes in HbA1c and body weight were investigated at two months, four months, and six months after the last administration of tirzepatide.

Biochemical analyses and instruments

After overnight fasting, each participant underwent a physical examination and venous blood collection. Body measurements were taken in the standing position. BMI was calculated as weight (kg) / height (m)^2^. Collected blood samples were centrifuged and assayed for plasma glucose and HbA1c. Plasma glucose levels were measured by the glucose oxidase method. HbA1c levels were measured by high-performance liquid chromatography (HPLC) (Q Squared Solutions Co. Ltd., Tokyo, Japan).

Statistical analysis

The data are expressed as the mean±standard error (S.E) or median (25th-75th percentile), depending on the data distribution or chart format. Differences in continuous variables between subcategories were tested for significance using analysis of covariance. Categorized variables were analyzed using the χ2 test. All analyses were performed using IBM SPSS Statistics for Windows, Version 29 (Released 2022; IBM Corp., Armonk, New York, United States).

## Results

Of the nine subjects (five men, four women), five subjects (three men, two women) were included in the tirzepatide 5 mg group, one subject (one man) was included in the tirzepatide 10 mg group, two subjects (two women) were included in the tirzepatide 15 mg group, and one subject (one man) was included in the dulaglutide 0.75 mg group (Table 1). Five patients were taking oral antidiabetic monotherapy and required washout before administration of active drugs: 3 metformin 500 mg/day, 1 metformin 2000 mg/day, and 1 empagliflozin 10 mg/day. Mean HbA1c across all cases was 7.9±0.3%, and the mean BMI was 33.5±3.3 kg/m^2^. In the tirzepatide 15 mg group, although no statistically significant difference was observed, average HbA1c was 7.3±0.2% and BMI 29.1±3.4 kg/m^2^, whose values were better compared to other groups.

**Table 1:** Baseline characteristics of participants BW, body weight; BMI, body mass index; FPG, fasting plasma glucose; HbA1c, hemoglobin A1c; GFR, glomerular filtration rate; SBP, systolic blood pressure; DBP, diastolic blood pressure; HR, heart rate; LDL, low-density lipoprotein; HDL, high-density lipoprotein. Washout, subjects who were taking a single antidiabetic drug before the start of the clinical trial and started the trial after stopping the drug. Data are presented as number, mean ± S.E., or median (25th‒75th percentile levels).

	Total	Dulaglutide 0.75mg	Tirzepatide 5mg	Tirzepatide 10mg	Tirzepatide 15mg
n	9	1	5	1	2
Sex, men /women	5 /4	1 /0	3 /2	1 /0	0 /2
Age, years	54.3 ± 5.4	79.0	46.6 ± 7.2	54.0	61.5 ± 5.5
BW, kg	91.8 ± 8.6	71.6	102.6 ± 12.4	104.8	68.2 ± 4.7
BMI, kg/m^2^	33.5 ± 3.3	24.9	36.3 ± 5.4	36.6	29.1 ± 3.4
FPG, mg/dL	145.6 ± 6.3	129.0	157.6 ± 6.3	147.0	123.0 ± 4.0
HbA1c, %	7.9 ± 0.3	7.4	8.3 ± 0.5	7.6	7.3 ± 0.2
eGFR,ml/min/1.73 m^2^	84 (57-98)	59.0	98 (67-100)	84.0	69
SBP, mmHg	139.2 ± 5.1	130.0	143.8 ± 5.5	139.0	143.0 ± 11.0
DBP, mmHg	89.3 ± 4.1	68.0	92.6 ± 4.8	100.0	86.5 ± 6.5
HR, bpm	72.4 ± 4.4	64.0	70.4 ± 6.8	74.0	81.0 ± 11.0
LDL-C, mg/dL	115.3 ± 9.4	143	106.4 ± 13.8	98	132.5 ± 15.5
Non HDL-C, mg/dL	139.4 ± 9.9	160	128.0 ± 16.3	149	153.0 ± 11.0
HDL-C, mg/dL	43.7 ± 2.0	56	42.8 ± 2.4	39	42.0 ± 0.0
Washout, yes/no	5/ 4	1/ 0	3/ 2	1/ 0	0/ 2

**Figure 1:**
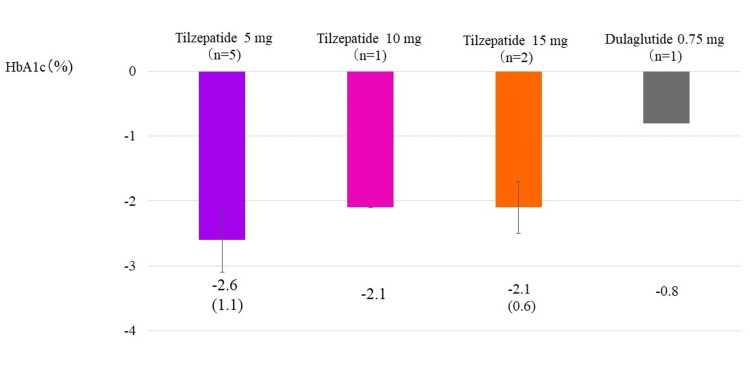
Change in HbA1c from the start of study drug administration to week 52 Change in HbA1c from the start of the study to week 52 in tirzepatide 5, 10, and 15 mg subgroups and the group receiving dulaglutide 0.75 mg, which is the primary outcome of the Surpass J-mono study [20]. The column and error bars indicate mean±standard error.

Figure 1 shows the change in HbA1c after 52 weeks, the primary outcome of the SURPASS J-mono study (Figure 1).

Average values were -2.6±1.1% in the tirzepatide 5 mg group, -2.1% in the tirzepatide 10 mg group, -2.1±0.6% in the tirzepatide 15 mg group, and -0.8% in the dulaglutide 0.75 mg group. No dose-dependent decrease in HbA1c was observed in the tirzepatide groups; this is presumed to be because mean HbA1c before initiation was relatively low in the tirzepatide 15 mg group, as mentioned above. After the start of tirzepatide administration, six patients experienced transient loss of appetite and nausea along with decreased intake of staple foods and snacks, but no patient discontinued the study drug due to severe gastrointestinal symptoms. One patient had a mild cutaneous injection site reaction. No hypoglycemia was observed in any participant during the study period.

After completing study drug administration, outpatient physicians resumed diabetes medications other than tirzepatide in some patients for glycemic control (Table 2).

**Table 2:** Resumption of antidiabetic drugs after trial completion in each group After study completion, outpatient physicians resumed diabetes medications in some patients for glycemic control.

		Resumed medication				
	Number	Two months later	Four months later	Six months later	One year later	Two years later
Tirzepatide 5 mg (n=5)	1	No drug resumption			Semaglutide 0.5 mg/week	
	5	No drug resumption				
	6	No drug resumption	Teneligliptin 20 mg/day			
	8	Metformin 1000 mg/day				Metformin 1000 mg/day＋Sitagliptin 25 mg/day
	12	No drug resumption				
Tirzepatide 10 mg (n=1)	11	No drug resumption	Metformin 500 mg/day			Metformin 500 mg/day ＋Dulaglutide 0.75 mg/week
Tirzepatide 15 mg (n=2)	2	No drug resumption				
	3	No drug resumption				
Dulaglutide 0.75 mg (n=1)	4	No drug resumption	Dulaglutide 0.75 mg/week			

**Figure 2:**
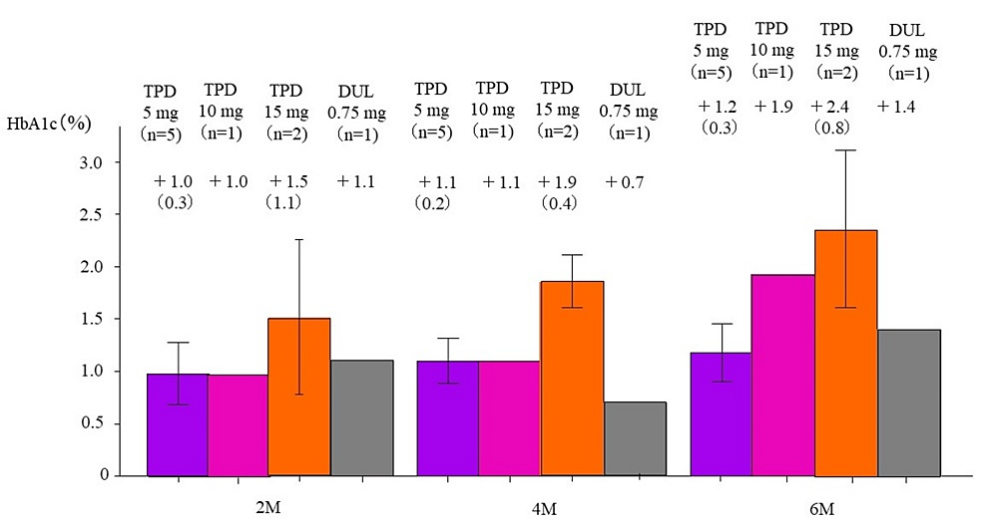
Change in HbA1c at two, four, and six months after drug discontinuation Change in HbA1c levels after study completion in the groups receiving 5 mg/10 mg/15 mg of tirzepatide and those receiving 0.75 mg dulaglutide. M, months; TPD, tirzepatide; DUL, dulaglutide. The column and error bars indicate mean±standard error.

At two, four, and six months after the end of study drug administration, respective HbA1c changes (%) were +1.0, +1.1, and +1.2 in the tirzepatide 5 mg group; +1.0, +1.1, and +1.9 in the 10 mg group; +1.5, +1.9, and +2.4 in the 15 mg group; and +1.1, +0.7, and +1.4 in the control group (Figure 2).

**Figure 3:**
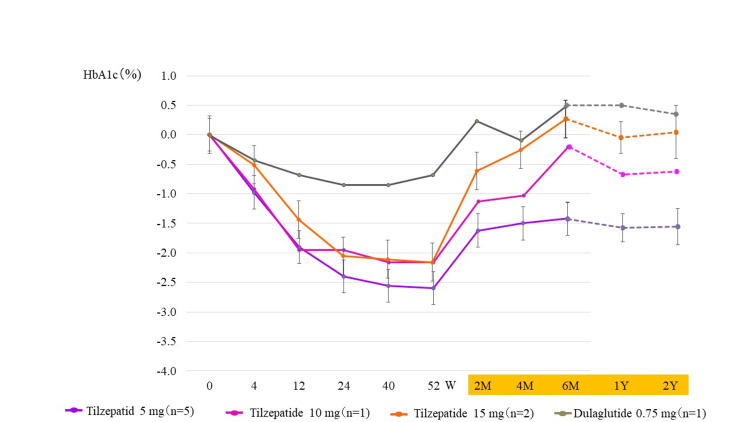
Change in HbA1c over the course of the trial and two years after drug discontinuation W, weeks; M, months; Y, year(s). The column and error bars indicate mean±standard error.

Figure 3 shows changes in HbA1c over the course of the trial and two years after drug discontinuation. Two months after discontinuing tirzepatide, re-elevation of HbA1c was observed (Figure 3). At one and two years after the end of study drug administration, respective HbA1c changes (%) were -1.7 and -1.6 in the tirzepatide 5 mg group; -1.2 and -1.1 in the 10 mg group; -0.1 and 0.0 in the 15 mg group; and +0.6 and +0.3 in the dulaglutide 0.75 mg group (Figure 3).

**Figure 4:**
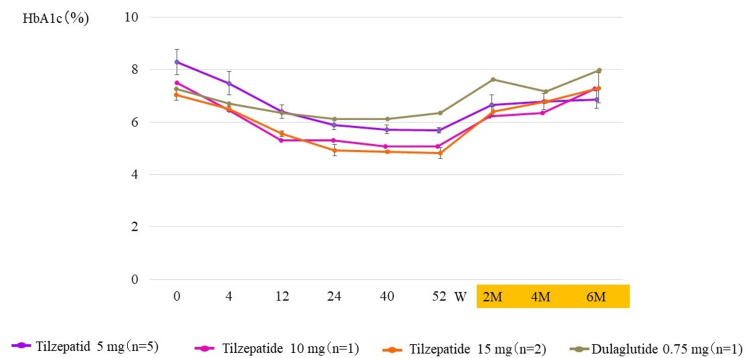
Change in HbA1c values over the course of the trial and two, four, and six months after drug discontinuation W, weeks; M, months. The column and error bars indicate mean±standard error.

The changes in HbA1c levels during the transition period are shown in Figure 4. Similar to the overall analysis of the SURPASS J-mono trial [20], the tirzepatide group achieved <6% HbA1c, but re-elevation was observed two months after the end of administration (Figure 4).

**Figure 5:**
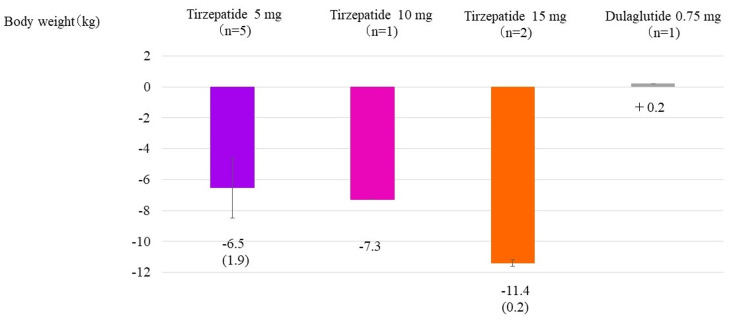
Change in body weight from the start of study drug administration to week 52 Change in body weight from the start of the study to week 52 in the tirzepatide 5, 10, and 15 mg subgroups and the group receiving 0.75 mg dulaglutide. The column and error bars indicate mean±standard error.

Body weight changes at week 52 were -6.5 ± 1.9 kg (6.0% decrease) in the tirzepatide 5 mg group, -7.3 kg (7.0% decrease) in the 10 mg group, and -11.4 ± 0.2 kg (16.8% decrease) in the 15 mg group. In contrast, the dulaglutide 0.75 mg group gained +0.2 kg (0.3% increase) (Figure 5).

**Figure 6:**
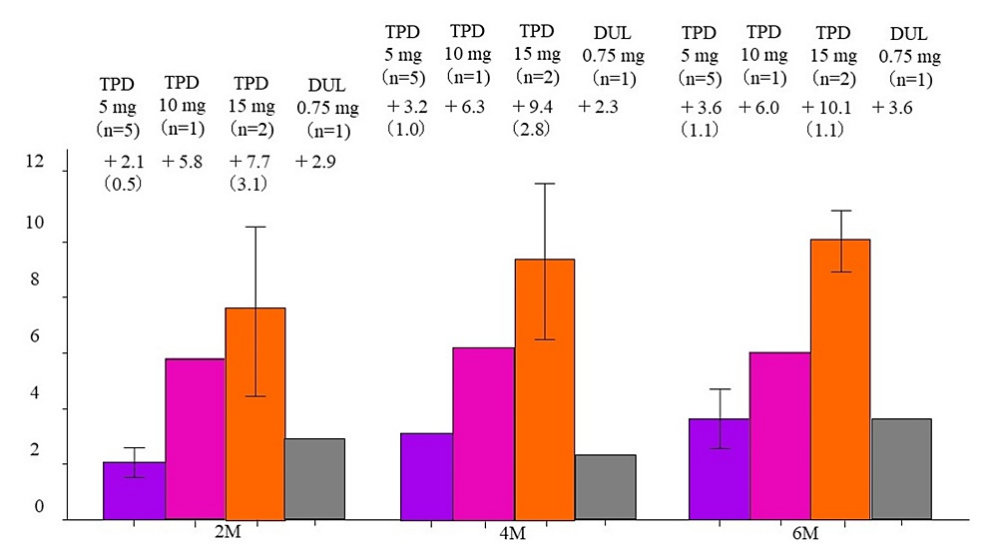
Change in body weight at two, four, and six months after drug discontinuation Change in body weight after study completion in the groups receiving 5 mg/10 mg/15 mg of tirzepatide and those receiving 0.75 mg dulaglutide. M, months; TPD, tirzepatide; DUL, dulaglutide. The column and error bars indicate mean±standard error.

At two, four, and six months after completion of study drug administration, the respective body weight changes were +2.1 kg (2.2% increase), +3.2 kg (3.2% increase), and +3.6 kg (3.6% increase) in the tirzepatide 5 mg group; +5.8 kg (6.0% increase), +6.3 kg (6.4% increase), and +6.0 kg (6.2% increase) in the 10 mg group; +7.7 kg (13.9% increase), +9.4 kg (17.0% increase), and +10.1 kg (18.0% increase) in the 15 mg group; and +2.9 kg (4.0% increase), +2.3 kg (3.2% increase), and +3.6 kg (5.0% increase) in the dulaglutide 0.75 mg group (Figure 6).

**Figure 7:**
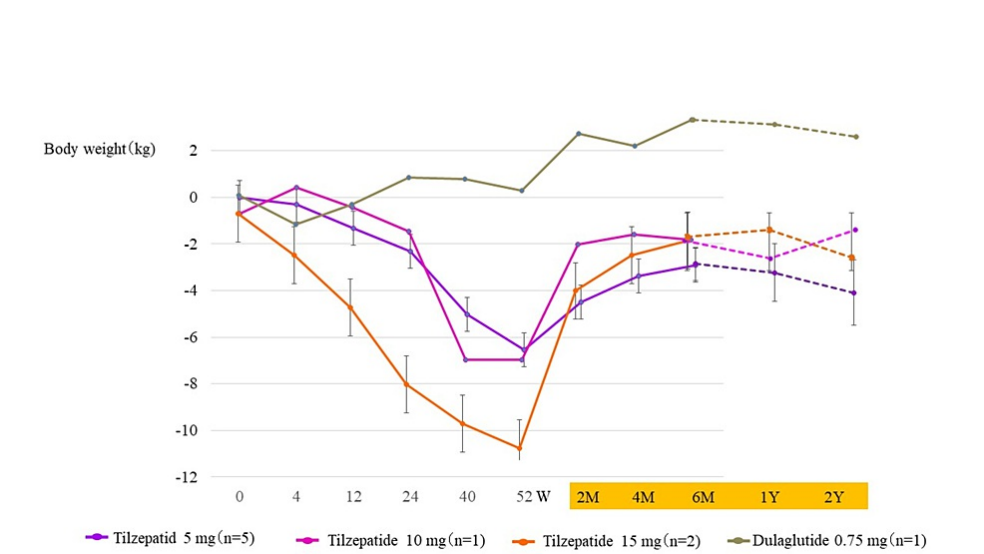
Change in body weight over the course of the trial and two years after drug discontinuation W, weeks; M, months; Y, year(s). The column and error bars indicate mean±standard error.

Figure 7 shows changes in body weight over the course of the trial and two years after drug discontinuation. Two months after discontinuing tirzepatide, weight regain was observed (Figure 7). Changes in body weight two years after drug discontinuation were -3.6 kg (3.5% decrease) and -5.1 kg (5.0% decrease) in the tirzepatide 5 mg group; -2.5 kg (2.3% decrease) and -0.6 kg (0.6% decrease) in the 10 mg group; -0.9 kg (1.3% decrease) and -2.6 kg (3.8% decrease) in the 15 mg group; and +3.4 kg (4.7% increase) and +2.5 kg (3.5% increase) in the dulaglutide 0.75 mg group (Figure 7).

**Figure 8:**
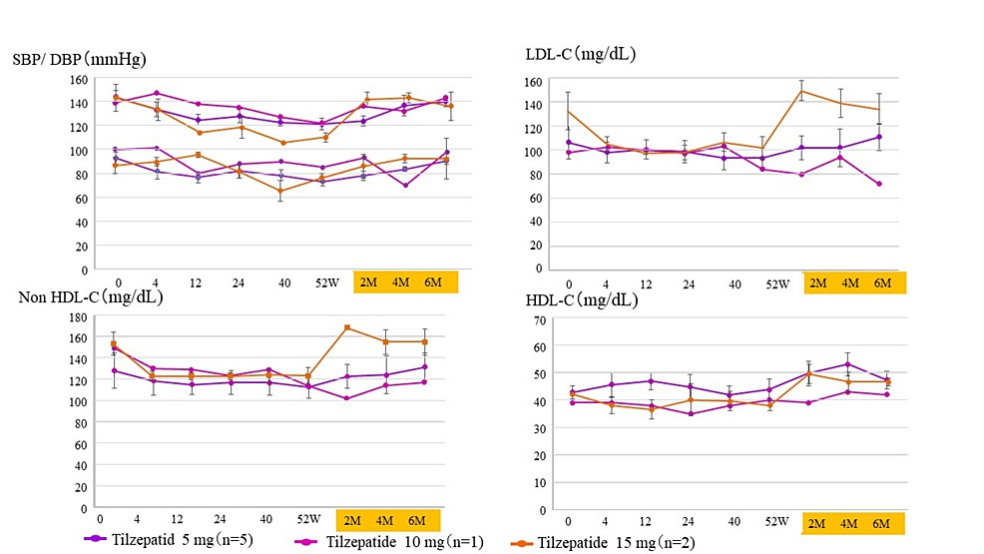
Progress of blood pressure and lipid metabolism markers in the tirzepatide group To assess the risk of arteriosclerosis after discontinuing tirzepatide, we examined changes in blood pressure and lipid metabolism markers during and up to six months after tirzepatide treatment. W, weeks; M, months. SBP, systolic blood pressure; DBP, diastolic blood pressure; LDL-C, low-density lipoprotein cholesterol; non-HDL-C, non-high-density lipoprotein cholesterol; HDL-C, high-density lipoprotein cholesterol; The column and error bars indicate mean±standard error.

Furthermore, we presented changes in blood pressure and lipid parameters after discontinuation of tirzepatide to evaluate the risk of atherosclerotic disease after discontinuation of tirzepatide (Figure 8). In all tirzepatide groups, blood pressure and lipid parameters improved during drug administration but tended to worsen when the drug was stopped.

## Discussion

In this study, compared with dulaglutide, tirzepatide exhibited an excellent effect on blood glucose improvement and dose-dependent weight loss, and the user experience was consistent with the overall results of the SURPASS J-mono study [20]. However, in this study, a rebound of exacerbated glucose tolerance and weight gain was observed relatively early after the end of tirzepatide administration. Although there has been another report of worsening blood glucose and weight control after discontinuing GLP-1 receptor agonists [23], this is the first report that describes in detail the changes in blood glucose and weight after discontinuing tirzepatide, a novel GIP and GLP-1 receptor dual agonist. Patients and their physicians often consider discontinuing or reducing antidiabetic drugs due to improved glycemic management; these findings suggest that strict lifestyle guidance and follow-up observation are necessary when stopping tirzepatide.

Tirzepatide has an effect on body weight [20,21]. In mice, endogenous GIP has a fat-storing effect [16], but administration of GIP at pharmacological concentrations has an appetite-suppressing and weight-reducing effect [17]. It has been shown that GIP action in the central nervous system, especially in the hypothalamus, may contribute to this weight loss effect [24]. Administration of GIP to high-fat-diet-treated mice increased blood levels of leptin, an anti-obesity hormone [25], while administration of tirzepatide in obese mice enhanced the influx of branched-chain amino acids into brown adipose tissue and stimulated thermogenic signaling [26]. In patients with type 2 diabetes, tirzepatide has been shown to delay gastric emptying, whose effect was most pronounced after the first dose and was attenuated by repeated doses [27]. In mice, tirzepatide may enhance peripheral energy metabolism [28]. Taken together, these reports indicate that tirzepatide acts on the appetite center of the central nervous system and presumably promotes a decrease in gastrointestinal peristalsis and an increase in activation of body metabolism, thereby leading to weight loss. Conversely, discontinuation of tirzepatide attenuates these effects, and a decrease in GIP receptor agonist activity after drug discontinuation may contribute to deterioration of glycemic control and rebound of weight gain. It has also been reported that weight loss can lead to increased chronic hunger due to increased ghrelin and decreased satiety caused by decreased leptin [29]. Furthermore, discontinuation of GLP-1 receptor agonists eliminates appetite suppression and alleviates gastrointestinal symptoms [23], as well as attenuation of glucose-dependent insulin secretion-stimulating and glucagon secretion-inhibiting effects [30]. Through these mechanisms, blood glucose and body weight rebound may occur. These reports and the results of this study suggest that clinicians should be careful about blood glucose and weight rebound when discontinuing tirzepatide after treatment of type 2 diabetes whose treatment management has once sufficiently improved with tirzepatide. This study showed that stopping tirzepatide immediately may not be advisable and after achieving breakthrough weight loss with tirzepatide, it may be difficult to maintain the loss through lifestyle and behavioral changes alone. Furthermore, it was also suggested that sudden discontinuation of tirzepatide may increase the once-reduced risk of atherosclerotic disease again (Figure 8).

In this study, a rapid rebound in the blood glucose level and body weight was observed in the tirzepatide group immediately after the end of the treatment, but in the long-term follow-up one and two years after discontinuation, slight improvement of HbA1c and body weight was observed compared to the start of the study (Figures 3, 7). This suggests that, even after the end of the clinical trial, many of the subjects who participated in this case series recognized more proactive lifestyle changes such as diet and exercise therapy because of their self-affirmation that they could successfully manage their diabetes with tirzepatide use for a period of time. In non-diabetic obese adults, the combination of liraglutide and exercise therapy was shown to be more effective than liraglutide alone in reducing rebound after weight loss treatment [31]. Therefore, combination therapy with exercise may be effective in suppressing blood glucose and body weight rebound after discontinuation of tirzepatide. Besides, appropriate reinstatement of oral hypoglycemic agents and GLP-1 receptor agonists by outpatient physicians, especially in the tirzepatide 5 mg and tirzepatide 10 mg groups, may have contributed to this result (Table 2).

Study limitations

There are some limitations of this research. First, this study was a case series study with a limited number of patients. A large number of reports of tirzepatide use in type 2 diabetes are needed to determine the effects after tirzepatide discontinuation. Second, this study was planned and analyzed at a single center independently of the SURPASS J-mono study. A multi-center randomized study is desirable to examine longer-term effects after discontinuation of tirzepatide. Third, post-trial effects, such as reduced frequency of medical interviews, may partially explain the rebound in blood glucose and weight following completion of the phase III trials of tirzepatide.

## Conclusions

This study suggests that it may be necessary to pay attention to rebound after stopping tirzepatide. In diabetes treatment, when aiming for remission, an exit strategy, that is, cessation or dose reduction of therapeutic drugs, should be considered. In patients with type 2 diabetes in need of weight loss, after improving glycemic control with the use of tirzepatide, either strict lifestyle guidance or follow-up, continued use of tirzepatide or use of alternative agents such as GLP-1 receptor agonist after its discontinuation is needed in order to maintain good diabetes management.
